# Retraction Notice: Effect of chest ultrasound compared with pericardial window for the diagnosis of occult penetrating cardiac wounds in hemodynamically stable subjects with penetrating thoracic trauma: A meta‐analysis

**DOI:** 10.1111/iwj.14276

**Published:** 2023-06-07

## Abstract

Yan, T, Xie, W, Xu, M. Effect of chest ultrasound compared with pericardial window for the diagnosis of occult penetrating cardiac wounds in hemodynamically stable subjects with penetrating thoracic trauma: A meta‐analysis. *International Wound Journal*. 2023; 1–9, https://doi.org/10.1111/iwj.14101. The above article from *International Wound Journal*, published online on January 30, 2023 in Wiley Online Library (wileyonlinelibrary.com) has been retracted by agreement between the journal Editor in Chief, Professor Keith Harding and John Wiley & Sons, Ltd. The retraction has been agreed due to unattributed overlap between this article and the following article: Manzano‐Nunes, A. Gomez, D. Espitia et al., A meta‐analysis of the diagnostic accuracy of chest ultrasound for the diagnosis of occult penetrating cardiac injuries in hemodynamically stable patients with penetrating thoracic trauma. *Journal of Trauma and Acute Care Surgery*, 2021, 90(2):p 388–395, https://doi.org/10.1097/TA.0000000000003006.

Yan, T, Xie, W, Xu, M. Effect of chest ultrasound compared with pericardial window for the diagnosis of occult penetrating cardiac wounds in hemodynamically stable subjects with penetrating thoracic trauma: A meta‐analysis. *International Wound Journal*. 2023; 1–9, https://doi.org/10.1111/iwj.14101. The above article from *International Wound Journal*, published online on January 30, 2023 in Wiley Online Library (wileyonlinelibrary.com) has been retracted by agreement between the journal Editor in Chief, Professor Keith Harding and John Wiley & Sons, Ltd. The retraction has been agreed due to unattributed overlap between this article and the following article: Manzano‐Nunes, A. Gomez, D. Espitia et al., A meta‐analysis of the diagnostic accuracy of chest ultrasound for the diagnosis of occult penetrating cardiac injuries in hemodynamically stable patients with penetrating thoracic trauma. *Journal of Trauma and Acute Care Surgery*, 2021, 90(2):p 388–395, https://doi.org/10.1097/TA.0000000000003006.

